# Identifying LATS2 as a prognostic biomarker relevant to immune infiltrates in human esophageal squamous cell carcinoma

**DOI:** 10.3389/fgene.2022.952528

**Published:** 2022-09-02

**Authors:** Minqi Zhu, Guoran Liao, Yuxuan Wang, Junxian Mo, Dunbo Yi, Yuhong Zhang, Lei Xian

**Affiliations:** ^1^ Department of Cardiothoracic Surgery, The Second Affiliated Hospital of Guangxi Medical University, Nanning, China; ^2^ Department of Cardio-Thoracic Surgery, The Seventh Affiliated Hospital of Guangxi Medical University, Wuzhou, Guangxi, China; ^3^ The First Affiliated Hospital of Guangxi Medical University, Nanning, China

**Keywords:** esophageal squamous cell carcinoma, overall survival, immune status, LATS2, ESCC

## Abstract

According to the TIMER database, large tumor suppressor 2 (LATS2) is differentially expressed in various tumors. However, the correlation between LATS2 and esophageal squamous cell carcinoma (ESCC) and the association between LATS2 and immune infiltration in ESCC remain unclear. Our synthetic research on LATS2 in ESCC revealed that the expression was low in esophageal squamous epithelium tissues, revealing the pernicious and adverse prognosis of ESCC. The Kaplan–Meier survival investigation pointed out that low LATS2 expression would result in an adverse prognosis. Biological investigation indicated that LATS2 was engaged in cell migration, adhesion, and junction. To further explore the relationship between LATS2 and tumor immunity, we utilized CIBERSORT to assess immune infiltration. The findings revealed that specimens with lower LATS2 expression showed higher immune infiltration, including T-cell follicular helper cells, M0 macrophages, M1 macrophages, and myeloid dendritic cell resting. An association investigation indicated that LATS2 was negatively relevant to immune checkpoints that restrain operative antitumor immune reactions. We also conducted immunohistochemical staining to explore the link between LATS2 expression and immunophenotype. The indicated association between low LATS2 expression and an immunophenotype is conducive to our understanding of ESCC mini-environments and might offer new indications for enhancing new therapeutic targets.

## Introduction

Esophageal cancer is the sixth principal cause of death worldwide. It manifests as a poor prognosis during diagnosis and a high mortality ratio. Its main subtypes are ESCC and adenocarcinoma, of which the former is the most commonly seen (Uhlenhopp et al., 2020). The operative early tumor markers and diagnostic approaches for esophageal squamous cell carcinoma are still insufficient. We have found a growing number of crucial driver genes as technology advances. Nevertheless, many unknown key genes remain, especially those related to the immune microenvironment of ESCC. Recently, as research on the Hippo signaling path has gradually deepened, we have found that it not only regulates the size of organs but also its dysregulation is closely related to tumor occurrence (Pan, 2010; Harvey et al., 2013; Meng et al., 2016). As a key kinase in the Hippo channel, LATS2 is prevalent in the current research (Avruch et al., 2012). However, many studies have revealed that LATS2 is low-expressed in some tumors. For instance, LATS2 is negatively correlated with the outcome of patients with gliomas (Shi et al., 2019). In addition, in osteosarcoma, microRNA-744 accelerates osteosarcoma progression by inhibiting LATS2 (Sun et al., 2019). Simultaneously, miR-103 fosters metastasis and EMT by targeting LATS2 in hepatocellular carcinoma (Han et al., 2018). There is evidence for the functional and clinical significance of LATS2 in ESCC pathogenesis and prognosis, revealing a close relationship between LATS2 and tumorigenesis (Gao et al., 2017).

Several bioinformatics approaches were used in this study to explore the relationship between LATS2 and ESCC and immune infiltration and its molecular regulation. LATS2 was low-expressed in ESCC, which may explain the shorter survival rate. In addition, LATS2 may be involved in cell migration (Yamaguchi and Condeelis, 2007; Duff and Long, 2017), adhesion (Läubli and Borsig, 2019), and connection (Wu et al., 2017; Gardiner and Cukierman, 2022), which are closely related to tumor occurrence and development. Additionally, we assessed the association between LATS2 expression and key molecules associated with the immune system. Finally, immunostaining was performed to determine the expression mode of LATS2 and its association with immune-related factors. These findings indicate that LATS2 may play a significant role in carcinogenesis and regulating immune cell infiltration in ESCC.

## Materials and approaches

### Tumor immune estimation Resource investigation

TIMER is considered a comprehensive resource that systematically investigates immune infiltrates covering various cancers (Li et al., 2016). The TIMER data bank can be employed to investigate 10,897 specimens covering 32 tumors in TCGA data bank (Li et al., 2017). The TIMER database is constructed of several modules. For example, the “DiffExp” model can help us explore the differential expression between tumors and normal neighboring tissues. We employed this model to analyze the association between mRNA expression levels of LATS2 with tumor and normal tissues.

### LATS2 expression in different cancers in ONCOMINE

The mRNA expression levels of LATS2 between normal and tumor tissues in various cancers were investigated in the ONCOMINE data bank (www.oncomine.org), TCGA, and GEO data. We obtained RNA-seq data (level 3) of esophageal carcinoma (ESCA) from TCGA data bank (https://portal.gdc.cancer.gov/), which covered 162 ESCA specimens. In addition, we obtained LATS2 expression data of 82 ESCC patients and 11 corresponding normal tissue specimens from TCGA database. We also extracted the patient’s clinical information. Simultaneously, we obtained data on LATS2 expression in normal esophageal tissues from GTEx V8 (https://gtexportal.org/home/datasets).

Subsequently, we searched for ESCC as a keyword in the GEO (www.ncbi.nlm.nih.gov/geo) warehouse to obtain potential microarray data. Then, we examined whether samples with ESCC tumors and normal tissues were in the selected datasets. Two tests were chosen in addition in the existing investigation: GSE23400 (Platform: GPL97) and GSE161533 (Platform: GPL570) (Su et al., 2011; Li et al., 2014; Hyland et al., 2016).

### Pre-processing procedures

We compiled the clinical information of 162 samples of ESCA from TCGA data bank and the relationship with LATS2 expression levels. Depending on the median of LATS2 gene expression, specimens are separated into two groups, namely, LATS2 high expression and LATS2 low expression. This step was carried out by introducing R software, whose version was 3.6.3. The original data were obtained from the GEO data bank. The extractive data were normalized and handled using log2 conversion. Probes were transformed into gene markers depending on the description information. The batch effect function was utilized as an original quality control procedure by utilizing variance stability count to remove individual horse influence. According to each dataset design, the specimens were separated into two groups: the normal one and the tumor one. We utilized GraphPad Prism 8.0 to map LATS2 gene expression differences in ESCC.

### Prognostic investigation

The original data and the matching clinical information of RNA sequence data (level 3) of 82 ESCC patients were obtained from TCGA. In addition, a timeROC investigation was performed to compare the forecasting precision and the risk level of LATS2. The analytical methods were carried out using R software, whose version was 3.6.3, and the “ggrisk” and “timeROC” R packages. The Kaplan–Meier Plotter instrument (www.kmplot.com) consists of survival data of 82 patients who suffered from ESCC (Nagy et al., 2021). Regarding Kaplan–Meier curves, p-values and hazard rate (HR) with a 95% confidence interval (CI) were produced by introducing log-rank tests and univariate Cox proportional hazard regression. In our investigation, all specimens were divided into groups with high and low expression based on the median mRNA expression. OS represents the time between diagnosis and death; HR represents the risk factor of the group with high expression relative to the low-expression group.

### Investigation of LATS2-interacting genes and proteins

The GeneMANIA database (http://www.genemania.org) was employed to build up LATS2 interaction network. The STRING database (https://string-db.org/) was employed to build a protein–protein interaction (PPI) network of LATS2.

### Correlation and enrichment analysis

An association investigation between LATS2 and other mRNAs in ESCC was conducted using TCGA data. We screened genes according to the following criteria from TCGA dataset: the result is cor value ≥ 0.5 and p value < 0.05. We took the top 50 genes for the correlation analysis. The correlation investigation was carried out by R software, whose version was 3.6.3, and the “ggstat” R package. The map was realized by the “ggplot2” R package. The functional enrichment analysis was utilized to investigate the data further to explore the underlying influence of potential targets. We screened positively correlated genes with LATS2 gene expression from TCGA dataset and selected the top 300 genes for enrichment investigation to mirror the function of LATS2. Gene Ontology (GO) investigation and Kyoto Encyclopedia of Genes and Genomes (KEGG) investigation were carried out by utilizing the enrichGO function in the “clusterProfiler” R package. The map was achieved by the “ggplot2” R package.

### Immune cell infiltration

To compare immune cell infiltration levels, we used CIBERSORT to score the immune cell infiltration in 82 ESCC patient tumor tissues and 11 paired adjacent tissues from TCGA database (Newman et al., 2015).

### Immunohistochemistry

ESCC and adjacent normal tissues (10 pairs) were obtained from The Second Affiliated Hospital of Guangxi Medical University (Guangxi, China). The study recruited patients aged 52 to 63 (52.2 ± 5.82714) from May 2019 to January 2020, half of them being males and half of them being females. The inclusion criteria included no prior history of any other active cancer, no active cancer treatment, and no history of esophageal cancer. The tissue samples were acquired by resection. The distance between ESCC tissues and adjacent normal tissues was about 1 cm. The Ethics Committee of The Second Affiliated Hospital of Guangxi Medical University (Guangxi, China) approved the use of human samples (approval no. 2021-0300). Written informed consent was obtained from all patients enrolled in the study. All specimens were used following the Ethics Committee’s protocol. The primary antibodies used were rabbit anti-LATS2 (20276-1-AP, Wuhan Sanying Biotechnology, Wuhan, China), rabbit anti-CTLA4 (53560, CST), and rabbit anti-PDL1 (13684, CST). The IOD/area ratio was calculated using Image-Pro Plus 6.0 and GraphPad Prism 8.0 for statistical analysis.

### Statistical analysis

TIMER plots were used to assess the statistical significance of various expressions using Wilcoxon rank-sum tests. In ONCOMINE, the results were displayed with *p*-values, fold changes, and ranks. TCGA boxplots were assessed with unpaired t-tests. Geo boxplots and immunohistochemistry pictures were assessed with a paired *t*-test. Kaplan–Meier plots were displayed with the HR and *p*-values from the log-rank test. Spearman’s correlation evaluated the correlation between gene expression and statistical significance. All results were considered to be statistically significant at *p* < 0.05. *: *p* < 0.05; **: *p* < 0.01; and ***: *p* < 0.001.

## Results

### The expression of LATS2 in pan-cancer

**FIGURE 1:**
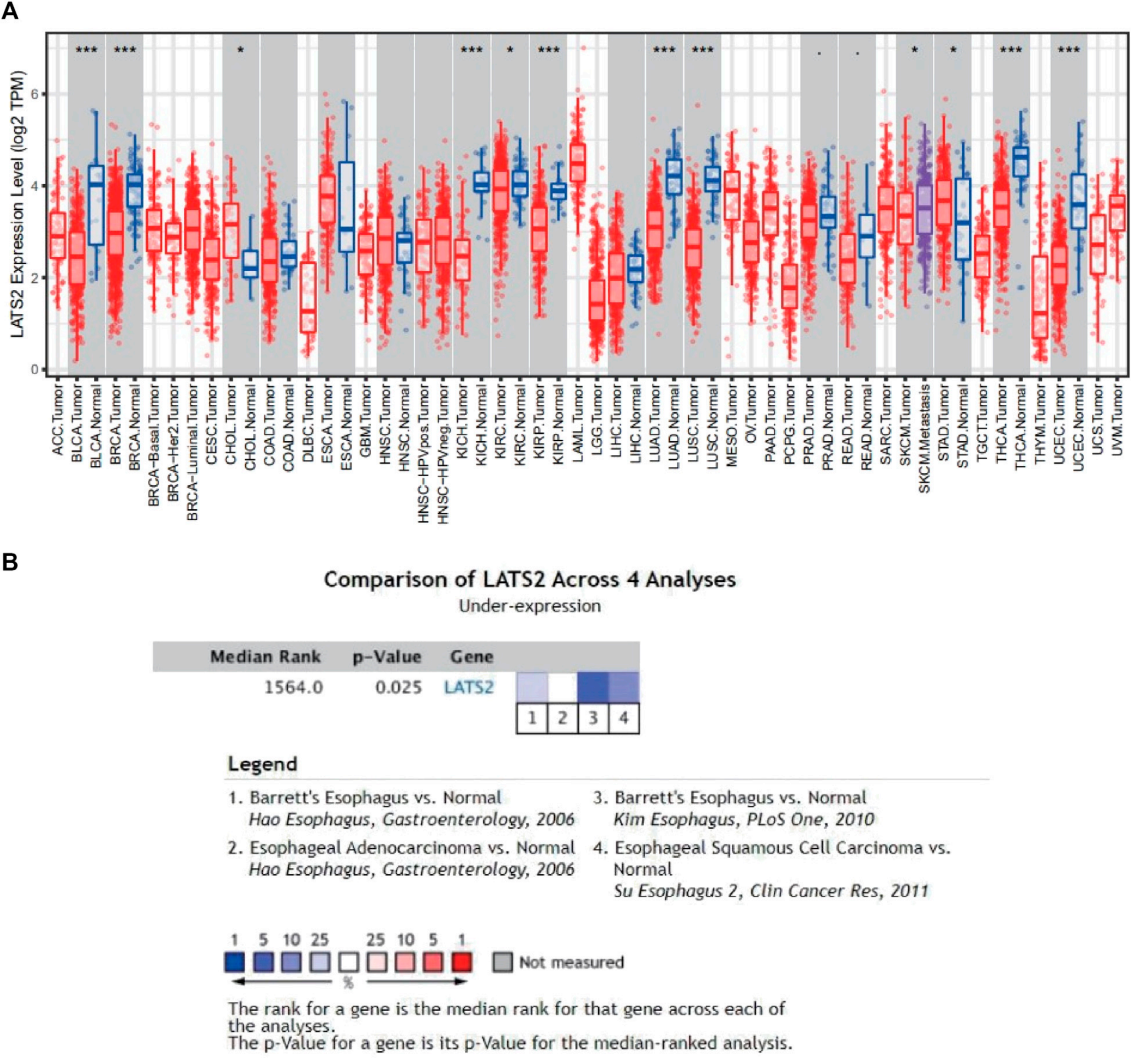
**(A)** Pan-cancer analysis expression of LATS2 based on the TIMER1.0 database. **(B)** Meta-analysis expression of LATS2 in ESCC in the ONCOMINE database.

We first utilized the data obtained from TCGA in the TIMER data bank to assess the mRNA expression of LATS2 in pan-cancer. The differences in LATS2 mRNA expressions between the tumor group and the normal group are displayed in Figure 1A. LATS2 mRNA expression in BLCA (urothelial bladder carcinoma), BRCA (invasive breast carcinoma), KICH (kidney chromophobe), LUAD (lung adenocarcinoma), LUSC (lung squamous cell carcinoma), and THCA (thyroid carcinoma) was lower than that in the normal tissues. To further confirm the relationship between LATS2 and ESCC, the ONCOMINE data bank was employed to analyze the mRNA expression level of LATS2 in the range of overall cancer. The findings showed that LATS2 expression in numerous cancer groups was lower than that in the corresponding normal groups. ONCOMINE database analysis also showed that the mRNA expression of LATS2 in ESCC was decreased compared with that in the normal group (Figure 1B).

### Association between LATS2 expression and clinicopathological factors

The 162 patient samples we collected from TCGA data bank were separated into two groups. First, the association between LATS2 expression and clinicopathological factors is presented in Table 1. We concluded that LATS2 expression was linked to race and histological type, especially in ESCC, LATS2 expression is lower (Table 1).

**TABLE 1:** Relationship between clinical factors and LATS2 expression in ESCC.

Characteristic	Level	Low expression of LATS2	High expression of LATS2	p
N		81	81	
T stage, n (%)	T1	15 (20.5%)	12 (16.7%)	0.268
	T2	19 (26%)	18 (25%)	
	T3	39 (53.4%)	38 (52.8%)	
	T4	0 (0%)	4 (5.6%)	
N stage, n (%)	N0	36 (49.3%)	30 (42.3%)	0.701
	N1	30 (41.1%)	33 (46.5%)	
	N2	5 (6.8%)	4 (5.6%)	
	N3	2 (2.7%)	4 (5.6%)	
M stage, n (%)	M0	61 (93.8%)	60 (93.8%)	1.000
	M1	4 (6.2%)	4 (6.2%)	
Pathologic stage, n (%)	Stage I	9 (12.5%)	7 (10%)	0.599
	Stage II	38 (52.8%)	31 (44.3%)	
	Stage III	21 (29.2%)	28 (40%)	
	Stage IV	4 (5.6%)	4 (5.7%)	
Radiation therapy, n (%)	No	52 (70.3%)	55 (78.6%)	0.343
	Yes	22 (29.7%)	15 (21.4%)	
Primary therapy outcome, n (%)	PD	5 (9.4%)	5 (12.2%)	0.836
	SD	3 (5.7%)	4 (9.8%)	
	PR	2 (3.8%)	1 (2.4%)	
	CR	43 (81.1%)	31 (75.6%)	
Gender, n (%)	Female	10 (12.3%)	13 (16%)	0.653
	Male	71 (87.7%)	68 (84%)	
Race, n (%)	Asian	22 (28.9%)	16 (23.5%)	0.035
	Black or African American	6 (7.9%)	0 (0%)	
	White	48 (63.2%)	52 (76.5%)	
Age, n (%)	≤60	41 (50.6%)	42 (51.9%)	1.000
	>60	40 (49.4%)	39 (48.1%)	
BMI, n (%)	≤25	44 (56.4%)	40 (53.3%)	0.826
	>25	34 (43.6%)	35 (46.7%)	
Histological type, n (%)	Adenocarcinoma	26 (32.1%)	54 (66.7%)	<0.001
	Squamous cell carcinoma	55 (67.9%)	27 (33.3%)	
Histologic grade, n (%)	G1	11 (16.7%)	5 (8.3%)	0.346
	G2	34 (51.5%)	32 (53.3%)	
	G3	21 (31.8%)	23 (38.3%)	
Smoker, n (%)	No	25 (34.2%)	22 (31%)	0.811
	Yes	48 (65.8%)	49 (69%)	
Alcohol history, n (%)	No	19 (24.4%)	27 (33.3%)	0.283
	Yes	59 (75.6%)	54 (66.7%)	
Barrett’s esophagus, n (%)	No	52 (82.5%)	54 (78.3%)	0.690
	Yes	11 (17.5%)	15 (21.7%)	
Tumor central location, n (%)	Distal	51 (63.7%)	62 (76.5%)	0.196
	Mid	25 (31.2%)	17 (21%)	
	Proximal	4 (5%)	2 (2.5%)	
Age, median (IQR)		60 (54, 70)	60 (53, 75)	0.519

### LATST2 expression in ESCC

**FIGURE 2:**
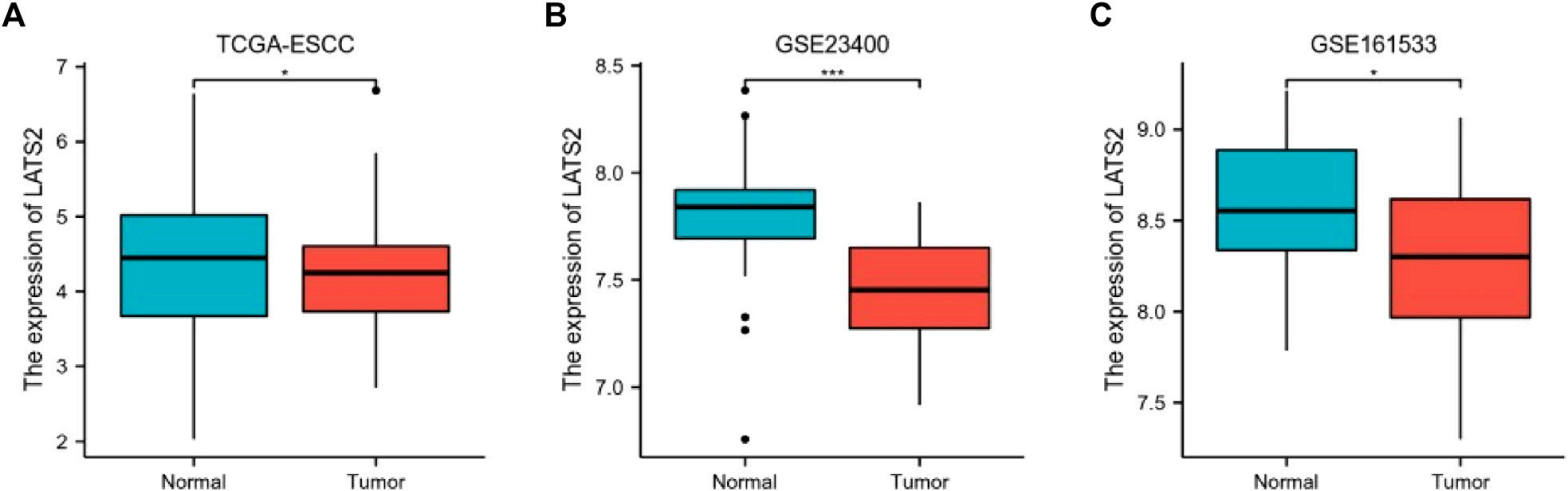
The differential expression of LATS2 in TCGA **(A)**, GSE23400 **(B)**, and GSE161533 **(C)** datasets.

The relationship between LATS2 expression and ESCC was further illustrated. The normalization and standardization datasets of GSE23400 and GSE161533 are depicted in Supplementary Figure. We concluded that the mRNA expression of LATS2 was lower in ESCC tissues than in normal esophageal tissues in all datasets (Figure 2). These findings revealed that a low level of LATS2 expression might represent the virulent evolution of ESCC.

### Association between LATS2 expression and cancer patient prognosis

**FIGURE 3:**
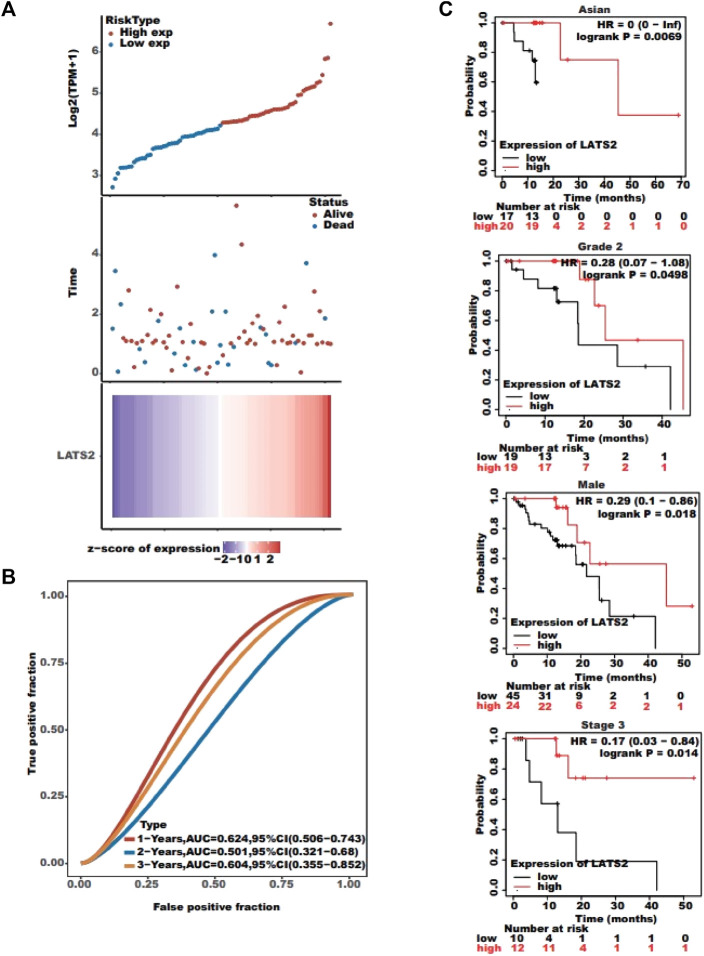
**(A)** Top graph represents the scatter plot of LATS2 expression from low to high; the middle graph refers to the scatter plot distribution of survival time and survival status corresponding to LATS2 gene expression in different samples; and the bottom graph represents the expression heat map of LATS2. **(B)** ROC curve and AUC assessed the performance of LATS2. **(C)** Kaplan–Meier survival analysis revealed that patients with increased LATS2 expression had longer OS in Asian, grade 2, male, and stage 3.

The association between LATS2 expression and survival time and survival status was evaluated using TCGA dataset. The top graph represents the scatter diagram of LATS2 expression and different colors representing different expression groups (Figure 3A); the middle graph refers to the scatter diagram allocation of survival time and situation matching to LATS2 gene expression in various specimens (Figure 3A); the basal graph refers to the expression heat chart of the gene (Figure 3A). The link between LATS2 expression and OS was investigated using TCGA cohort to assess the value of LATS2 during cancer patient prediction. The Kaplan–Meier survival investigation revealed that patients who suffered from increased LATS2 expression had longer OS in Asian, grade 2, male, and stage 3 (Figure 3C). Furthermore, the ROC curve and AUC values at different times for LATS2 gene were calculated, wherein AUC values should be between 0 and 1; the better the model, the higher the prediction effect, that is, when the model randomness is 0.5, the AUC prognosis model should typically be at least 0.7 (Figure 3B). These findings proved that a high LATS2 expression had a good prognosis in patients with ESCC, and the gene expression level could be utilized to forecast OS efficiently.

### Identification of LATS2-interacting genes and proteins

**FIGURE 4:**
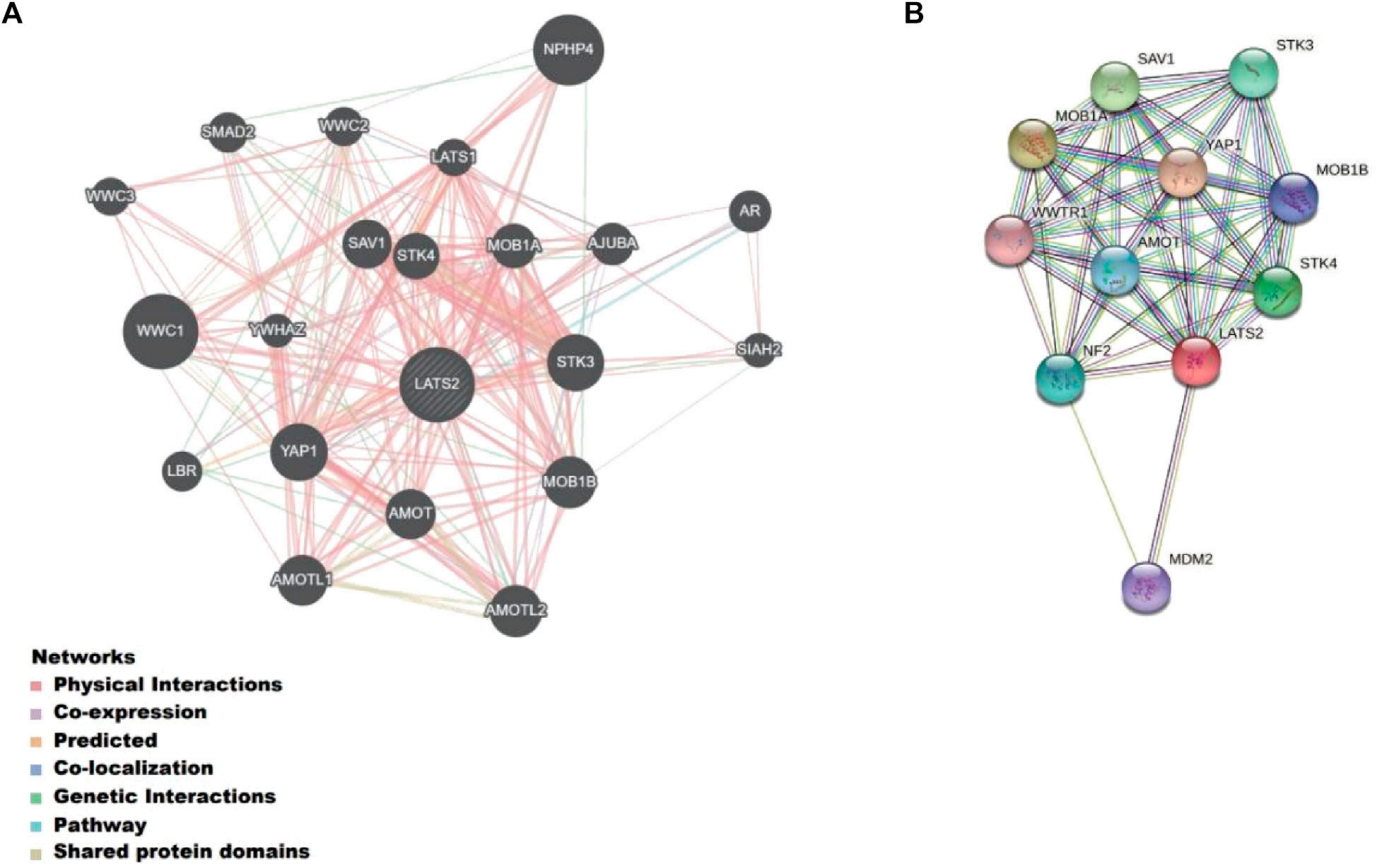
**(A)** Gene–gene interaction network for LATS2 and the altered neighboring genes constructed on GeneMANIA. **(B)** Protein–protein interaction identification on the STRING database.

We built a gene–gene interaction network for LATS2 and the altered adjacent genes with GeneMANIA (Figure 4A). The findings indicated that the 20 genes that changed most frequently were relevant to LATS2, such as YAP1 (Shibata et al., 2018), STK3 (Wang et al., 2020), STK4 (Lin et al., 2020), AMOT (Ruan et al., 2016), and MOB1A (Praskova et al., 2008). In addition, the PPI network of LATS2 was produced using the STRING data bank (Figure 4B). These results indicated that LATS2 may be crucial in tumor inhibition by limiting cell propagation and enhancing apoptosis.

### Correlation and enrichment analyses

**FIGURE 5:**
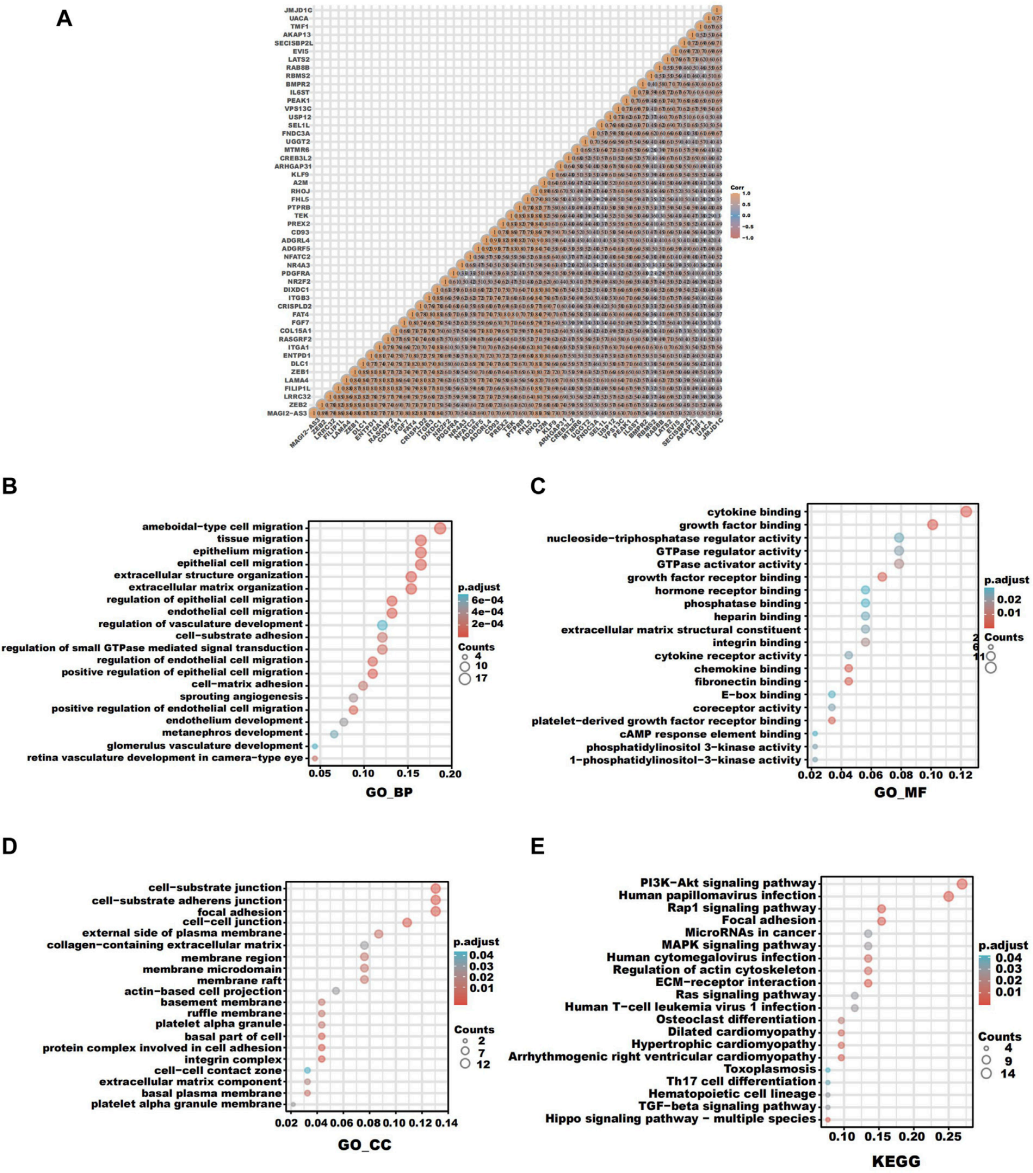
**(A)** Correlation analysis between LATS2 and other genes in ESCC. **(B)** GO enrichment analysis about the biological process. **(C)** GO enrichment analysis about molecular function. **(D)** GO enrichment analysis about cellular components. **(E)** KEGG enrichment analysis.

To forecast the role of LATS2 as well as the relevant path, we carried out an association investigation between LATS2 and other kinds of genes in ESCC by employing TCGA data (Figure 5A). The top 100 genes that were most positively related to LATS2 were chosen for an enrichment investigation. The GO enrichment investigation showed the processes targeted by these expressed genes: epithelial cell migration and extracellular matrix organization (Figure 5B). The Gene Ontology molecular function includes cytokine binding, growth factor binding, chemokine binding, and fibronectin binding (Figure 5C). The Gene Ontology cellular components include cell–substrate adherens junction, a protein complex involved in cell adhesion, and an integrin complex (Figure 5D). The pathway enrichment analysis (KEGG) revealed that the PI3K-Akt signaling pathway, human papillomavirus infection, and ECM–receptor interaction were enriched (Figure 5E). These findings revealed that LATS2 is linked to many pathways associated with malignancy in ESCC, particularly in tumorigenesis.

### Correlation between immune cell infiltration and LATS2

**FIGURE 6:**
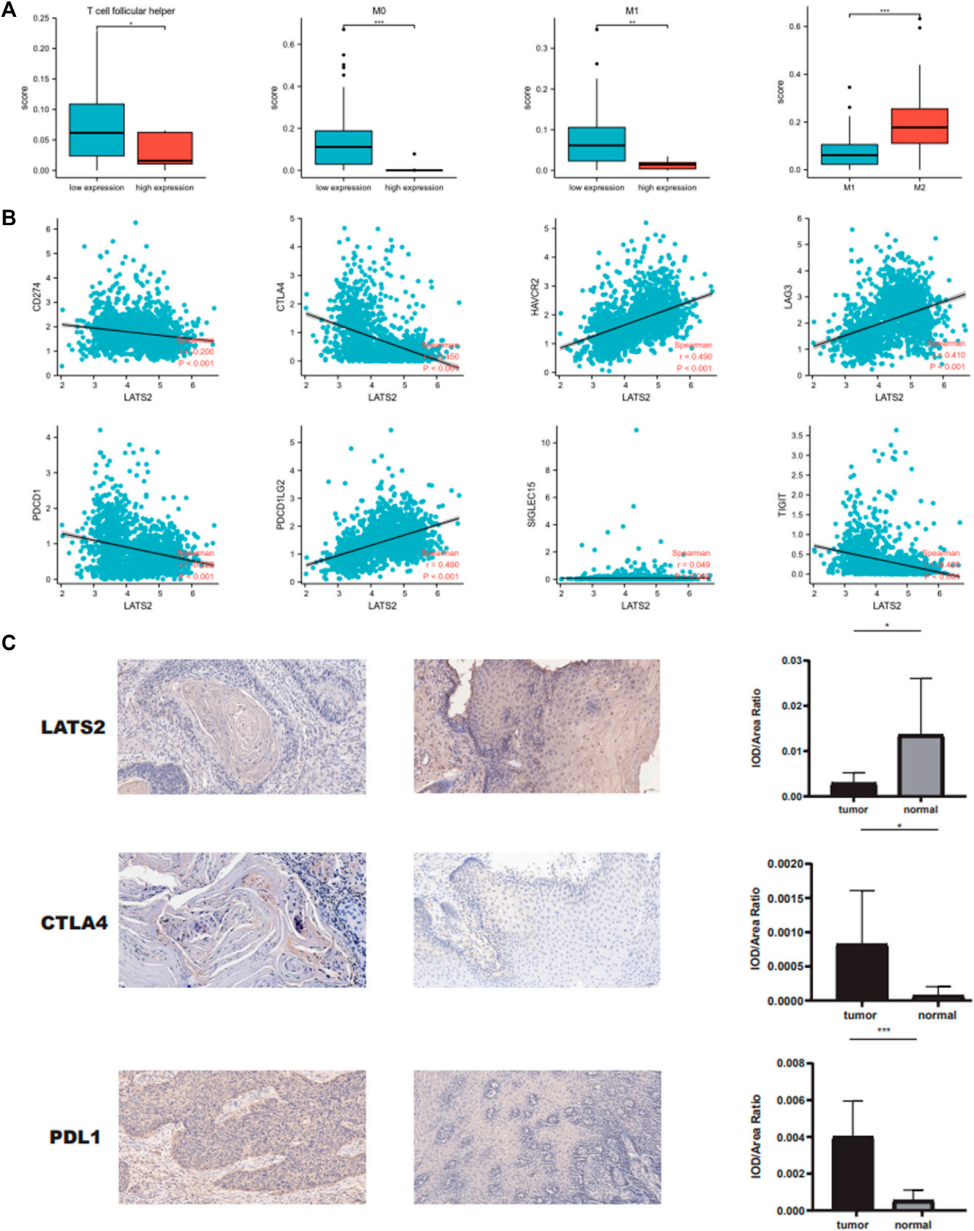
**(A)** LATS2 expression correlated with numbers of immune cells. **(B)** Correlation between LATS2 expression and immune checkpoints. **(C)** IHC staining of LATS2, CTLA4, and PD‐L1 in ESCC samples.

We carried out further research to assess the immune cell infiltration point of TCGA esophageal squamous cell carcinoma and found that samples with low LATS2 expression indicated large quantities of immune cells such as T-cell follicular helper M1 macrophages and myeloid dendritic cell resting (Figure 6A). Subsequently, we assessed the association of LATS2 expression with several significant immune checkpoints, which were able to mirror the immune mini-environment of ESCC with diverse LATS2 expression levels. Furthermore, LATS2 expression was negatively related to molecules that restrain the antineoplastic immune reaction, including TIGIT, PDCD1, CTLA4, and CD274 (Figure 6B). To demonstrate the link between LATS2 expression and the immunophenotype, ESCC specimens that showed low and high LATS2 expression levels were treated with immunohistochemical staining of CTLA4 and PD‐L1. As described in Figure 6C, the specimens with low LATS2 expression showed higher CTLA4 and PD‐L1 staining levels. Such findings might account for the disappointing prognosis of ESCC patients with a low level of LATS2 expression.

### Prognostic investigation of LATS2 expression based on immune cells in ESCC patients

**FIGURE 7:**
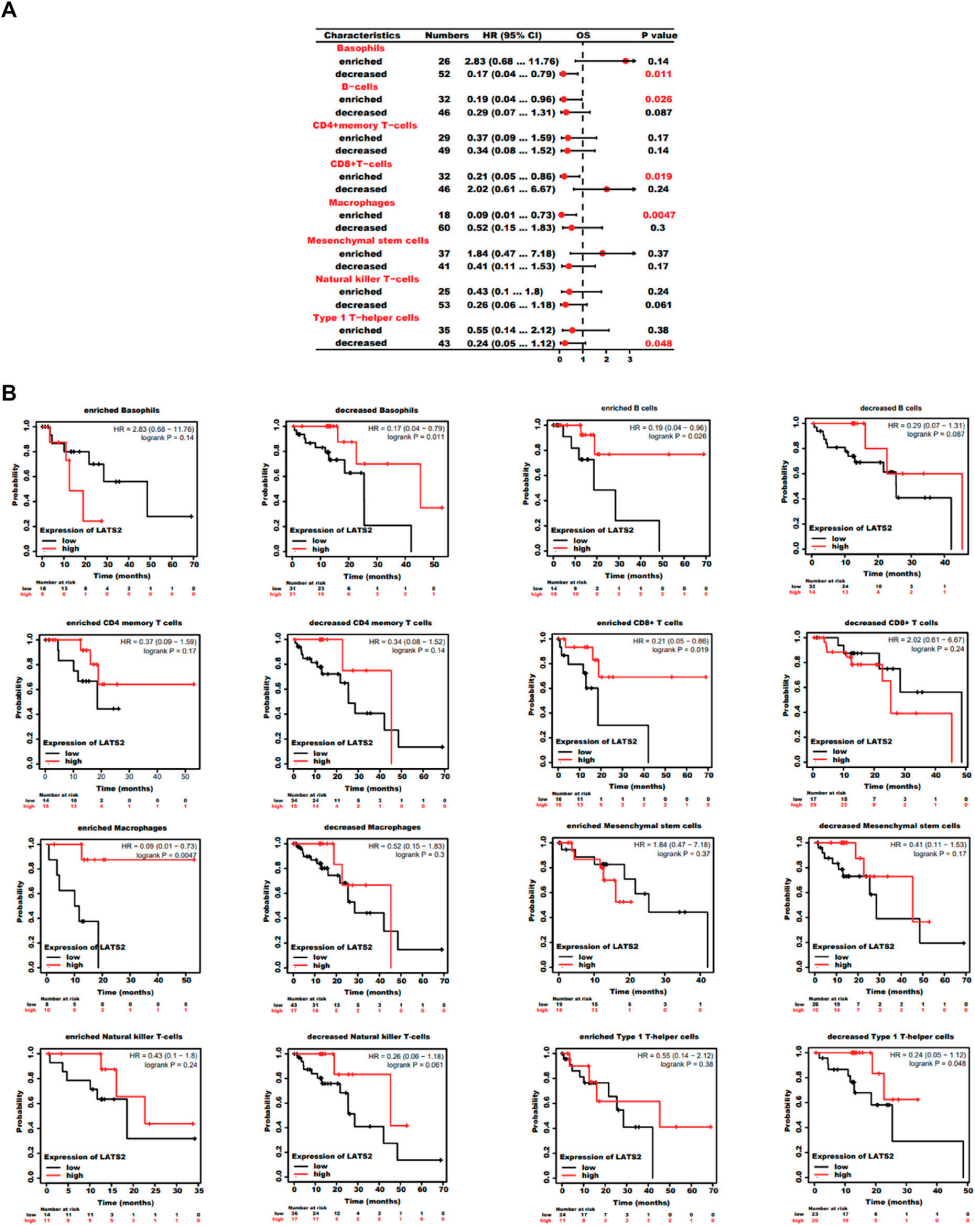
**(A)** Forest plot shows the prognostic value of LATS2 expression according to different immune cell subgroups in ESCC patients. **(B)** Kaplan–Meier plot was used to estimate the correlation between LATS2 expression and OS in different immune cell subgroups of ESCC patients.

As LATS2 expression is relevant to immune infiltration and the disappointing prediction in ESCC, we researched to find whether LATS2 expression would influence the prediction of ESCC due to immune infiltration. We conducted a prediction investigation based on the expression levels of LATS2 in ESCC in immune cell subgroups, which were associated, as displayed in Figures 7A,B. ESCC patients with low expression of LATS2 and descending infiltration of basophils and Th1 cells showed a descending prediction (Figures 7A,B). Nevertheless, there was a high association between LATS2 expression and the favorable prediction of ESCC in the group, which had increased infiltration of B cells, CD8^+^T cells, eosinophils, and macrophages. Such findings revealed that LATS2 may influence ESCC patients’ prediction to some extent because of immune infiltration.

## Discussion

Among malignant tumors, esophageal cancer is regarded as the eighth most commonly diagnosed cancer worldwide. At present, ESCC remains the most predominant kind globally. Despite progress in incipient diagnosis and immune treatment, esophageal squamous cell carcinoma is often detected at a later stage with a disappointing prediction (Uhlenhopp et al., 2020). The treatment of many diseases covers LATS2. Nevertheless, it has not been widely researched in tumors. Consequently, it is necessary to determine the function of LATS2 in predicting and treating cancer. Simultaneously, it has been studied in other tumors. However, there are few studies on LATS2 in ESCC. Therefore, it is necessary to determine the function of LATS2 in predicting and treating ESCC. Regarding the current investigation, we indicated that LATS2 expression in ESCC was lower than that in normal ones through bioinformatics investigation using ONCOMINE.

Furthermore, LATS2 was lowly expressed in ESCC in GSE161533 and GSE23400 data banks. After that, the clinical predictive importance of LATS2 in ESCC patients was explored, through which we found that the low expression of LATS2 was relevant to sex, age, and metastasis for ESCC patients. Such findings revealed that LATS2 may be regarded as a potent symbol for predicting patients with tumors and may enhance the targeted accuracy of oncology.

Published research studies on LATS2 have been mainly performed on glioma, osteosarcoma, and hepatocellular carcinoma. Our studies revealed an association between LATS2 and ESCC. GO findings revealed that LATS2 was linked to epithelial cell migration, cytokine binding, chemokine binding, fibronectin-binding, and cell–substrate adherens junction. On the other hand, KEGG investigation revealed that LATS2 was associated with human papillomavirus infection and ECM–receptor interaction. This may reveal that LATS2 may be relevant to the existence and progression of tumors in ESCC. The experimental results of Gao et al. (2017) are consistent with our predictions. The results of prognosis reports indicate that OS with low expression of LATS2 is poor, which may be related to some aspects of the immune microenvironment in ESCC (Gajewski et al., 2006; Zheng et al., 2020). Tumor mini-environment immune cells are a crucial factor in tumor tissues as more and more signals indicate their clinicopathological importance in forecasting survival conditions. Our findings indicated that ESCC with lower LATS2 expression were more infiltrated by M0 and M1 macrophages, while the penetration by CD8^+^T cells showed no increase. Simultaneously, in the LATS2 low-expression group, we found that M2 macrophages infiltrated more than M1 macrophages (Zheng et al., 2020). According to previous reports, the immune-inhibited cell populations were abundant in ESCC, including depleted CD8^+^T cells and M2 macrophages. According to a research study, undifferentiated M0 macrophages can generally diverge into typically activated macrophages (M1) with the pro-inflammatory/antitumoral phenotype. Furthermore, it can diverge into alternatively activated macrophages (M2) with the immune-inhibited/pro-tumoral phenotype (Vitale et al., 2019). Our results indicate that M1/M2 macrophages coexist in ESCC. The co-existence of M1 and M2 revealed that tumor-associated macrophages (TAMs) were more complicated than the typical modes (Zheng et al., 2020). To comprehend the tumor immunosuppression of ESCC, we made predictions through bioinformatics and verified them through IHC. The results confirmed that the tumor immunosuppressive environment of ESCC patients might be related to the relationship between LATS2 and PDL1 (Taube et al., 2014) and CTLA4 (Camacho, 2015). The results may provide a new direction for tumor immunotherapy for ESCC patients (Park et al., 2018; Zayac and Almhanna, 2020). In summary, LATS2 may occupy a significant position in immune cell infiltration and may be considered a valuable predictive symbol for ESCC. In tumor immunotherapy for ESCC patients, patients with high expression of LATS2 may benefit more from this treatment.

The existing investigation enhances our knowledge of the association between LATS2 and ESCC. However, its limitations should also be presented. First, though we have researched the association between LATS2 and immune infiltration for patients who suffer from ESCC, there is a lack of interpretation of immune analysis according to subgroups. Second, based on the present study, most investigations examined the protein levels of LATS2. A further investigation, supplemented on mRNA levels, made the findings more persuasive. In a word, our findings revealed that LATS2 could act as a potential and new predictive symbol for ESCC. Furthermore, we researched the potential evidence that LATS2 could adjust immune cell infiltration for patients who suffer from ESCC. As a result, such findings have underlying value in advancing our current understanding of the function of LATS2 and its translational application in ESCC prediction and immunization therapy.

In conclusion, LATS2 may be related to the occurrence, development, and prognosis of ESCC, and LATS2 may play an important role in the immune infiltration of ESCC, which provides potential value for the role of LATS2 in immunotherapy of ESCC.

## Data Availability

The original contributions presented in the study are included in the article/Supplementary Material; further inquiries can be directed to the corresponding author.
