# Supplementary figures and images for: Identifying LATS2 as a prognostic biomarker relevant to immune infiltrates in human esophageal squamous cell carcinoma
Source: Front Genet. 2022 Sep 2;13:952528. doi: 10.3389/fgene.2022.952528 (PMC9479129; doi:10.3389/fgene.2022.952528)

A

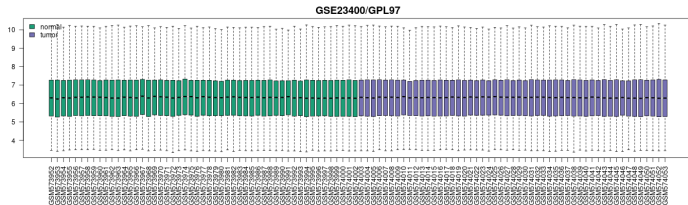

B

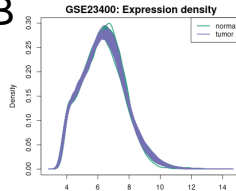

C

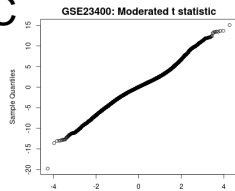

D

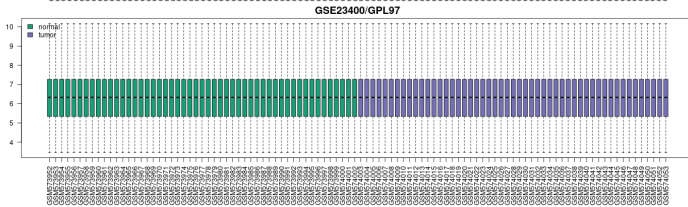

E

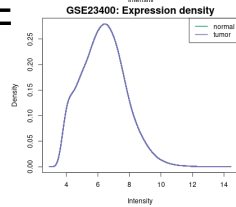

F

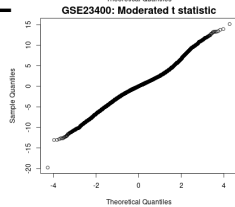

Supplement: Supplementary file 1 [file DataSheet1.PDF]
